# Prevalence and characteristics of patients with upper urinary tract urothelial carcinoma having potential Lynch syndrome identified by immunohistochemical universal screening and Amsterdam criteria II

**DOI:** 10.1186/s12885-023-11460-7

**Published:** 2023-10-05

**Authors:** Kenji Tanabe, Yasukazu Nakanishi, Naoya Okubo, Shunya Matsumoto, Yosuke Umino, Madoka Kataoka, Shugo Yajima, Teruhiko Yoshida, Saori Miyazaki, Takeshi Kuwata, Genichiro Ishii, Reiko Watanabe, Hitoshi Masuda

**Affiliations:** 1 Department of Urology, National Cancer Center Hospital East, 6-5-1 Kashiwanoha, Kashiwa-Shi, Chiba, 277–8577 Japan; 2 Department of Genetic Medicine, National Cancer Center Hospital, Tokyo, Japan; 3 Department of Pathology and Clinical Laboratories, National Cancer Center Hospital East, Chiba, Japan; 4 Department of Genetic Medicine, National Cancer Center Hospital East, Chiba, Japan; 5 Department of Pathology, St. Marianna University School of Medicine, Kanagawa, Japan

**Keywords:** Immunohistochemistry, Lynch syndrome, Surgery, Universal screening, Urothelial carcinoma

## Abstract

**Background:**

This study aimed to identify patients with upper urinary tract urothelial carcinoma (UTUC) having potential Lynch syndrome (pLS) by immunohistochemistry (IHC) of DNA mismatch repair gene-related proteins (MMRPs) and Amsterdam criteria II and explore their clinical characteristics.

**Methods:**

We retrospectively collected the clinical data of 150 consecutive patients with UTUC who underwent surgical resection at our institution between February 2012 and December 2020, and immunohistochemistry (IHC) of four MMRPs (*MLH1*, *MSH2*, *MSH6*, and *PMS2*) on all UTUC specimens was performed. Patients who tested positive for Amsterdam criteria (AMS) II and/or IHC screening were classified as having pLS and others as non-pLS, and their characteristics were explored.

**Results:**

In this study, 5 (3%) and 6 (4%) patients were positive for AMS II and IHC screening, respectively. Two patient were positive for both AMS II and IHC screening, resulting in 9 (6%) patients with pLS. The pLS group was predominantly female (67% vs. 36%; *p* = 0.0093) and had more right-sided tumors (100% vs. 43%; *p* = 0.0009) than the non-pLS group. Of the 6 patients who were positive for IHC screening, 4 showed a combined loss of *MSH2*/*MSH6* (*n* = 3) and *MLH1*/*PMS2* (*n* = 1). Other two patients showed single loss of *MSH6* and *PSM2*.

**Conclusions:**

AMS II and IHC screening identified pLS in 6% of patients with UTUC. The IHC screening-positive group tends to have relatively high rate of combined loss, but some patients have single loss. AMS II may overlook patients with LS, and a universal screening may be required for patients with UTUC as well as those with colorectal and endometrial cancer.

## Background

Lynch syndrome (LS) is an autosomal dominant hereditary disorder that increases the risk of various malignant diseases [1]. Among cancers that may develop in patients with LS during their lifetime, upper urinary tract urothelial carcinoma (UTUC) is the third most common cancer following colorectal and endometrial cancers [2, 3]. The diagnosis of LS is confirmed by genetic testing; however, it is costly, and patients with LS-related cancers generally undergo screening tests first. Screening tests consisted of primary screening with Amsterdam criteria (AMS) II (Table 1) [4] and secondary screening with immunohistochemistry (IHC) and/or microsatellite instability (MSI) [5]. However, because AMS II had a low sensitivity for the diagnosis of LS [6], universal screening with IHC and/or MSI, having high sensitivity for the diagnosis of LS, is recommended for all patients with colorectal and endometrial cancers [7, 8].

**Table 1:** Amsterdam criteria II for Lynch syndrome

*Positive when all the following are met*
*• There should be at least three relatives with any Lynch syndrome-related cancer (colorectal, endometrial, small bowel, ureteral, or renal pelvic cancer)*
*• One should be a first-degree relative of the other two*
*• At least two successive generations should be affected*
*• At least one should be diagnosed before the age of 50*
*• Familial adenomatous polyposis should be excluded in the colorectal cancer case(s), if any*
*• Tumors should be verified by pathological examination*

IHC confirms the loss of expression of proteins associated with the four DNA mismatch repair genes (MMR: *MLH1*, *MSH2*, *MSH6*, and *PMS2*) that are known to cause LS [9, 10]. The loss of MMR-related proteins (MMRPs) was reported in 90% of patients with LS-related UTUC [11]; however, universal screening for LS in patients with UTUC is not commonly performed. Metcalfe et al. uniquely performed universal screening for LS in patients with UTUC and referral of screening-positive patients to a genetic medicine department, resulting in 13.9% of screening-positive patients and 5.2% of patients diagnosed with LS [12]. Currently, the AUA and EAU guidelines recommend universal screening for LS in patients with UTUC. However, the benefit of this approach has not been validated enough, and more data are required. In this retrospective study, we performed IHC on all UTUC specimens from patients who had undergone surgical resection at our institution to identify patients who had potential LS (pLS) in combination with AMS II and explore their characteristics.

## Methods

From the medical records, we retrospectively collected the demographic, clinical, and pathological characteristics and oncological outcomes of 150 consecutive patients with UTUC who underwent surgical resection at our institution between February 2012 and December 2020. Five patients underwent segmental ureteral resection. A history of LS-related cancers of the patients and their relatives, including cancer of the colorectum, endometrium, small bowel, ureter, or renal pelvis, was reviewed to identify patients who were positive for AMS II (Table 1). The 8th edition of The International Union Against Cancer was used for the clinical staging of tumors. The pathological stages were reviewed by specialized urology pathologists at our institution.

IHC was performed on all 150 UTUC specimens that had been preserved in formalin-fixed and paraffin-embedded blocks to confirm the loss of MMRPs (IHC screening). The standard avidin–biotin–peroxidase complex method with an automated immunostainer (BenchMark ULTRA; Ventana Medical Systems, Tucson, AZ, USA) was used. The antibodies applied were MLH1 (M1), MSH2 (G219-1129), MSH6 (SP93), and PMS2 (A16-4) (all from Ventana Medical Systems). MMRP loss was determined by three specialized urology pathologists at our institution. When the tumor showed no nuclear expression in ≥ 95% of tumor cells and clear nuclear expression in background control cells in at least one MMRP, the case was positive for IHC screening.

Patients who tested positive and negative for AMS II and/or IHC screening were assigned to the pLS group and non-pLS group, respectively. The differences between groups were analyzed using the chi-square test or Fisher’s exact test for categorical variables and the Wilcoxon–Mann–Whitney U test for continuous data. All survival outcomes were calculated using the Kaplan–Meier method and compared using the log-rank test. Statistical significance was set at *p*-values of < 0.05. All statistical analyses were performed using the JMP software (SAS Institute Inc., version 13.2).

The institution’s ethics committee approved the study protocol, which complied with the provisions of the Declaration of Helsinki (Ethics Review Committee of our Institution, Research Project No. 2021–073). An opt-out method was applied to obtain consent for this retrospective study.

## Results

The patient, tumor, and pathological characteristics of the 150 patients are summarized in Table 2. Moreover, 5 (3%) and 6 (4%) patients were positive for AMS II and IHC screening, respectively. Two patients were positive for both AMS II and IHC screening, resulting in 9 (6%) patients with pLS. The AMS II-positive group had more family history of LS-related cancer (100 vs. 33%; *p* = 0.0047) and right-sided tumors (100 vs. 45%; *p* = 0.020) than the negative group. The IHC screening-positive group was predominantly women (83% vs. 26%; *p* = 0.0025) and had more right-sided tumors (100% vs. 44%; *p* = 0.0075) than the negative group. A history of LS-related cancers was likely to be more common in the IHC screening-positive group (33% vs. 9%; *p* = 0.051), and a trend toward differences in clinical T stage was found (*p* = 0.054). The pLS group was predominantly women (67% vs. 36%; *p* = 0.0093) and had more right-sided tumors (100% vs. 43%; *p* = 0.0009) than the non-pLS group.

**Table 2:** Patient, tumor, and pathological characteristics and screening results

		Amsterdam Criteria II			IHC screening ^a^			Potential LS ^b^		
	Total (*N* = 150)	Positive (*N* = 5)	Negative (*N* = 145)	*P* value	Positive (*N* = 6)	Negative (*N* = 144)	*P* value	Positive (*N* = 9)	Negative (*N* = 141)	*P* value
Patient characteristics										
Age (years), median (IQR)	72 (68–77)	67 (57–77)	72 (68–77)	0.26	74 (70–83)	72 (68–78)	0.39	74 (66–82)	73 (68–78)	0.86
Sex, n (%)				0.14			0.0025			0.0093
Male	107 (71)	2 (40)	105 (72)		1 (17)	106 (74)		3 (33)	104 (74)	
Female	43 (29)	3 (60)	40 (28)		5 (83)	38 (26)		6 (67)	37 (26)	
ECOG PS, n (%)				0.39			0.52			0.17
0	136 (91)	4 (80)	132 (91)		5 (83)	131 (91)		7 (78)	129 (91)	
1	14 (9)	1 (20)	13 (9)		1 (17)	13 (9)		2 (22)	12 (9)	
Charlson comorbidity index, n (%)				0.35			0.71			0.37
0	92 (61)	5 (100)	87 (60)		5 (83)	87 (60)		8 (89)	84 (59)	
1–2	49 (33)	0 (0)	49 (34)		1 (17)	48 (33)		1 (11)	48 (34)	
3–4	8 (5)	0 (0)	8 (5)		0 (0)	8 (6)		0 (0)	8 (6)	
≥ 5	1 (1)	0 (0)	1 (1)		0 (0)	1 (1)		0 (0)	1 (1)	
Smoking, n (%)				0.32			0.63			0.65
Never	45 (30)	3 (60)	42 (29)		2 (33)	43 (30)		3 (33)	42 (30)	
Ever or current	103 (69)	2 (40)	101 (70)		3 (50)	100 (69)		5 (56)	98 (69)	
Unknown	2 (1)	0 (0)	2 (1)		1 (17)	1 (1)		1 (11)	1 (1)	
Bladder cancer, n (%)				0.66			0.79			0.74
Never	107 (71)	4 (80)	103 (71)		4 (67)	103 (72)		6 (67)	101 (72)	
Ever or current	43 (29)	1 (20)	42 (29)		2 (33)	1 (28)		3 (33)	40 (28)	
History of LS-related cancer ^c^, n (%)				0.078			0.051			0.20
No	135 (90)	3 (60)	132 (91)		4 (67)	131 (91)		7 (78)	128 (91)	
Yes	15 (10)	2 (40)	13 (9)		2 (33)	13 (9)		2 (22)	13 (9)	
Family history of LS-related cancer				0.0047			0.30			0.057
No	90 (60)	0 (0)	90 (62)		2 (33)	88 (61)		2 (22)	88 (63)	
Yes	53 (35)	5 (100)	48 (33)		3 (50)	50 (35)		6 (67)	47 (33)	
Unknown	7 (5)	0 (0)	7 (5)		1 (17)	6 (4)		1 (11)	6 (4)	
Tumor characteristics										
Laterality, n (%)				0.020			0.0075			0.0009
Right	70 (47)	5 (100)	65 (45)		6 (100)	64 (44)		9 (100)	61 (43)	
Left	80 (53)	0 (0)	80 (55)		0 (0)	80 (56)		0 (0)	80 (57)	
Region, n (%)				0.65			0.59			0.97
Renal pelvis	66 (44)	3 (60)	63 (43)		2 (33)	64 (44)		4 (44)	62 (44)	
Ureter	84 (56)	2 (40)	82 (57)		4 (67)	80 (56)		5 (56)	79 (56)	
Clinical T stage, n (%)				0.69			0.054			0.19
a or 1	66 (44)	1 (20)	65 (45)		3 (50)	63 (44)		3 (33)	63 (45)	
2	22 (15)	1 (20)	21 (14)		1 (17)	21 (15)		2 (23)	20 (14)	
3	59 (39)	3 (60)	56 (39)		1 (17)	58 (40)		3 (33)	56 (40)	
4	3 (2)	0 (0)	3 (2)		1 (17)	2 (1)		1 (11)	2 (1)	
Clinical N stage, n (%)				0.71			0.53			0.69
0	133 (88)	5 (100)	128 (88)		5 (83)	128 (89)		8 (89)	125 (89)	
1	7 (5)	0 (0)	7 (5)		0 (0)	1 (5)		0 (0)	7 (5)	
2	10 (7)	0 (0)	10 (7)		1 (17)	9 (6)		1 (11)	9 (6)	
Pathological characteristics										
Number of tumors, n (%)				0.35			0.31			0.21
Single	129 (86)	5 (100)	124 (86)		6 (100)	123 (85)		9 (100)	120 (93)	
Multiple	21 (14)	0 (0)	21 (14)		0 (0)	21 (15)		0 (0)	9 (7)	
Gross appearance, n (%)				0.11			0.39			0.15
Papillary	101 (67)	5 (100)	96 (66)		5 (83)	96 (67)		8 (89)	93 (66)	
Non-papillary	49 (33)	0 (0)	49 (34)		1 (17)	47 (33)		1 (11)	48 (34)	
Histological features (primary), n (%)				0.85			0.83			0.79
Urothelial carcinoma	149 (99)	5 (100)	144 (99)		6 (100)	143 (99)		9 (100)	140 (99)	
Sarcoma	1 (1)	0 (0)	1 (1)		0 (0)	1 (1)		0 (0)	1 (1)	
Histological features (secondary), n (%)				0.90			0.13			0.42
None	135 (90)	5 (100)	130 (90)		4 (67)	131 (91)		7 (78)	128 (91)	
Squamous cell carcinoma	12 (8)	0 (0)	12 (8)		2 (33)	10 (7)		2 (22)	10 (7)	
Adenocarcinoma	2 (1)	0 (0)	2 (1)		0 (0)	2 (1)		0 (0)	2 (1)	
Urothelial carcinoma	1 (1)	0 (0)	1 (1)		0 (0)	1 (1)		0 (0)	1 (1)	
Tumor grade, n (%)				0.95			0.79			0.70
1	2 (1)	0 (0)	2 (1)		0 (0)	2 (1)		0 (0)	2 (1)	
2	113 (76)	4 (80)	109 (76)		4 (67)	109 (76)		6 (67)	107 (76)	
3	34 (23)	1 (20)	3 (23)		2 (33)	32 (23)		3 (33)	31 (22)	
Pathological T stage, n (%)				0.74			0.50			0.31
CIS	7 (5)	0 (0)	7 (5)		0 (0)	7 (5)		0 (0)	7 (5)	
a or 1	51 (34)	3 (60)	48 (33)		4 (66)	47 (33)		6 (67)	45 (32)	
2	26 (17)	1 (20)	25 (17)		1 (16)	25 (17)		1 (11)	25 (18)	
3	65 (43)	1 (20)	64 (44)		1 (16)	64 (44)		2 (22)	63 (44)	
4	1 (1)	0 (0)	1 (1)		0 (0)	1 (1)		0 (0)	1 (1)	
With CIS, n (%)				0.079			0.83			0.33
No	94 (63)	5 (100)	89 (61)		4 (67)	90 (62)		7 (78)	87 (62)	
Yes	56 (37)	0 (0)	56 (39)		2 (33)	54 (38)		2 (22)	54 (38)	
Lymphatic vessel invasion, n (%)				0.075			0.27			0.086
No	93 (62)	5 (100)	88 (61)		5 (83)	88 (61)		8 (89)	85 (60)	
Yes	57 (38)	0 (0)	57 (39)		1 (17)	56 (39)		1 (11)	56 (40)	

*CIS* carcinoma in situ, *ECOG PS* Eastern Cooperative Oncology Group Performance Status, *IHC* immunohistochemistry, *IQR* interquartile range, *LS* Lynch Syndrome

^a^IHC screening confirmed the expression of mismatch repair gene-related proteins (MMRPs). When any MMRP was deficient, IHC screening was positive

^b^Potential LS was defined as testing positive for Amsterdam Criteria II and/or MMR-related protein defect

^c^LS-related cancer includes colorectal, endometrial, small bowel, ureteral, or renal pelvic cancer

**Fig. 1:**
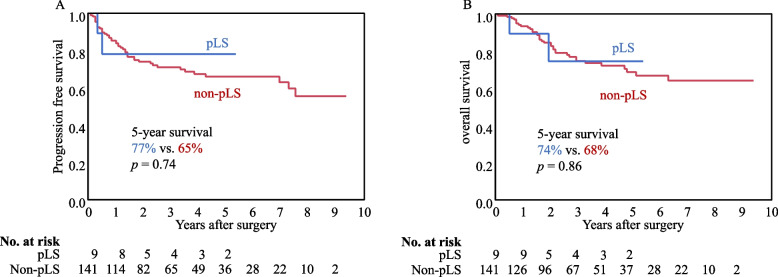
Survival after surgery was classified as pLS and non-pLS. (A) Progression-free survival and (B) overall survival. pLS, potential Lynch syndrome

As regards oncologic outcomes, the 5-year progression-free survival and overall survival rates were 77% and 74% in the pLS group, which did not significantly differ from those in the non-pLS group (65%, *p* = 0.74; 68%, *p* = 0.86) (Fig. 1).

**Fig. 2:**
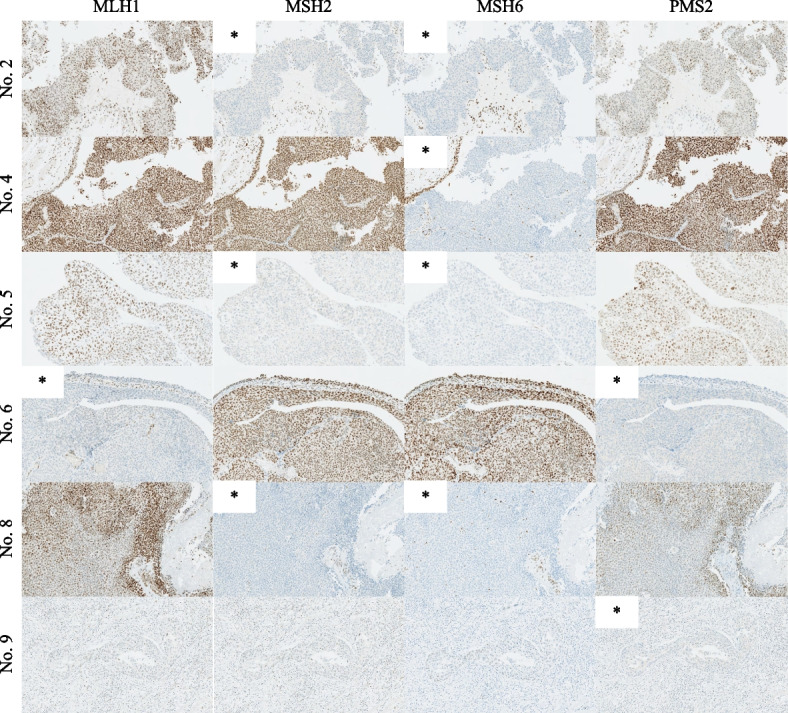
Immunohistochemistry results for four DNA mismatch repair gene-related proteins (100 × magnification). The number represents the patient number in Table 3. * Loss of protein expression

Table 3 summarizes the characteristics of nine patients with pLS. One patient was 48 years old, and the others were over 65 years old. Three patients were male, and six were female. Three patients had a history of LS-related cancer, in which two had colorectal cancer and one had endometrial cancer. Of the six patients in the IHC-screening-positive group, three had a loss of *MSH2* and *MSH6* gene-related proteins (Patients 2, 5, and 8), the other three had a loss in each of *MLH1* and *PMS2* (No. 6), *MSH6* alone (No. 4), and *PSM2* alone gene-related proteins (No. 9), respectively, and their staining diagram is shown in Fig. 2. Two patients who was positive for both AMS II and IHC screening had loss of *MSH2* and *MSH6,* and *MLH1* and *PMS2* gene-related proteins, respectively. (No.2 and 6 in Table 3, respectively).

**Table 3:** Individual results in patients with potential Lynch syndrome who tested positive in the screening

No	Age	Sex	History of LS-related cancer	Amsterdam Criteria II	MMRP loss	Genetic mutation
1	48	Male	No	Positive	Negative	Not done
2	65	Female	Colorectal	Positive	*MSH2*/*MSH6*	Not done
3	67	Male	No	Positive	Negative	Not done
4	71	Male	No	Negative	MSH6	Not done
5	73	Female	Endometrial	Negative	*MSH2*/*MSH6*	Not done
6	74	Female	Colorectal	Positive	*MLH1*/*PMS2*	*MLH1*
7	80	Female	No	Positive	Negative	Not done
8	83	Female	No	Negative	*MSH2*/*MSH6*	Not done
9	84	Female	No	Negative	*PSM2*	Not done

*LS* Lynch syndrome, *MMRP* mismatch repair gene-related protein

We have evaluated a case with a genetic mutation. A 74-year-old woman with a history of colorectal cancer was referred to the genetic medicine department and underwent genetic testing to confirm the diagnosis of LS with *MLH1* mutation (No. 6 in Table 3). The patient was diagnosed with colorectal cancer at the age of 68, and her mother was diagnosed with rectal cancer at the age of 41 and her aunt had gastric cancer in her 30 s. The patient was positive for AMS II, and MLH1 and PMS2 gene-related proteins were loss on IHC. The patient provided informed consent for the multi-institutional clinical genomics study (Protocol No. 2013–303, approved by the research ethics committee).

## Discussion

This study clarified the incidence of pLS in UTUC in a single Asian race. AMS II and IHC screening for LS in 150 patients with UTUC showed that 9 (6%) had pLS. Of the nine patients with pLS, five were positive for AMS II and six were positive for IHC screening, and two were positive for both AMS II and IHC screening. Despite the limitation of the small number of comparisons, no difference in prognosis was found between the groups.

Previous studies have reported that the clinicopathological characteristics of patients with UTUC in the pLS group were not significantly different from those in the non-pLS group, except for the age at diagnosis [13, 14]. With respect to age at diagnosis, some studies have reported that the pLS group was younger than the non-pLS group, whereas others reported no significant difference [12, 15]. On the contrary, studies in patients with UTUC diagnosed with LS (patients with LS-related UTUC) reported that LS-related UTUC was diagnosed at a younger age, more common in women, more likely to occur in the ureter, and more likely to be bilateral than sporadic UTUC [16–18]. In this study, the pLS group was predominantly female and had more right-sided tumors than the non-pLS group; however, the age at diagnosis did not differ significantly [13, 14, 19].

Several screening results for LS in patients with UTUC have been reported; however, the screening-positive rate was relatively low (2%–13%), and a few of these patients underwent genetic testing [12, 13]. In response to these reports, the AUA and EAU guidelines recommend that universal screening tests for LS be performed in patients with UTUC, but sufficient evidence has not been established, and screening for LS in patients with UTUC is not widely available. This report indicates the presence of no small number of pLS patients in the general population of UTUC patients and may provide a basis for recommending universal screening. AMS II was positive in 3%–8% of patients with UTUC, like colorectal cancer [12, 20, 21]. AMS II was reported to have a sensitivity of 22%–42% and specificity of 98% for LS diagnosis in a study of LS-related colorectal cancer [6, 19]. Because of the low sensitivity, universal screening with IHC and/or MSI instead of AMS II became widespread in colorectal and endometrial cancer [7, 8]. In UTUC, AMS II was reported to be positive in 66% of the patients diagnosed with LS by genetic testing [12]. On the contrary, 66% of the patients with a negative genetic test for LS were positive for ASM II [12]. In this study, 3% of the patients were positive for AMS II, and one of whom was diagnosed with LS. AMS II may be not sensitive enough to screen for LS in patients with UTUC, and universal screening using IHC might be required, as in patients with colorectal and endometrial cancers.

IHC screening was positive in 2%–11% of UTUC tumors, which was comparable with the results of universal screening in colorectal cancer [12, 14, 22, 23]. The most common pattern of MMRP loss observed in IHC screening of UTUC tumors was a combination of *MSH2* and *MSH6*, followed by *MSH2* alone, *MSH6* alone, and a combination of *MLH1* and *PMS2* [16]. A single *PMS2*loss was also reported [24]. Our study showed similar results; among the six patients in the IHC-positive group, three had a loss in *MSH2* and *MSH6* gene-related proteins, and the other three had a loss in each of *MLH1* and *PMS2*, *MSH6* alone, and *PSM2*alone gene-related proteins. IHC screening was reported to have a sensitivity of 83%–93% and a specificity of 89% for LS diagnosis in a study of LS-related colorectal cancer [19, 25]. In UTUC, some studies have reported IHC screening results followed by genetic testing. Metcalfe et al. reported that nine patients underwent genetic testing and six had a diagnosis of LS. Of the nine patients, two had false-positive IHC screening results and none had false-negative IHC screening results [12, 16]. Ito et al. screened 164 patients with UTUC, identifying positive in 4 (2%) patients. Three of these patients underwent genetic testing, all of whom had a diagnosis of LS. [13]

Reports of colorectal cancer have indicated that patients with a loss of *MHS2* or *MSH6* related-proteins in IHC screening were more likely to have LS-related colorectal cancer, whereas cases with loss of *MLH1* or *PSM2*related-proteins were more likely to have sporadic colorectal cancer [7]. On the contrary, patients with LS and germline *MLH1* or *MSH2* mutations in genetic testing had a higher lifetime incidence of LS-related malignant disease than patients with LS and germline *MSH6* or *PSM2*mutations [7]. In UTUC, although genetic test results are rarely reported, mutations in *MHS2* are the most common, followed by *MLH1*, *MSH6*, and *PSM2* [16]. In the present study, one patient diagnosed with LS had a loss of *MLH1* and *PSM2* gene-related proteins in IHC screening and was diagnosed as LS with a germline *MLH1* mutation in genetic testing.

This study has several limitations. First, this is a single-institution retrospective study with a small number of patients having pLS, which may lead to biases. Second, MSI was not assessed. However, Metcalfe et al. reported that MSI assessment was not necessary to identify pLS in patients with UTUC because no patients tested negative in IHC screening among patients with high MSI. [12] In addition, a study in colorectal cancer reported that the concordance between IHC screening and MSI was 97.5%, which led the NCCN guidelines to recommend that either IHC screening or MSI testing was sufficient for universal screening for LS [7, 25]. Third, most patients with pLS did not undergo genetic testing. Because this was a retrospective study, genetic testing could not be performed on patients who had already died or were no longer being followed up for various reasons. Owing to the unique characteristics of this test, it is up to the patient to decide whether to undergo genetic testing. Urologists must understand that this test requires sufficient informed consent, considering the ethical considerations and emotional burden on the patient.

## Conclusions

AMS II and IHC screening identified pLS in 6% of patients with UTUC. One patient was diagnosed with LS by genetic testing, whose AMS II and IHC screening were true positive. The IHC screening-positive group tends to have relatively high rate of combined loss, but some patients have single loss. AMS II may overlook patients with LS, and a universal screening may be required for patients with UTUC as well as those with colorectal and endometrial cancer.

## Data Availability

The datasets used and/or analysed during the current study available from the corresponding author on reasonable request.

## Abbreviations

AMS: Amsterdam criteria
IHC: Immunohistochemistry
LS: Lynch syndrome
MMR: DNA mismatch repair gene
MMRP: DNA mismatch repair gene-related protein
MSI: Microsatellite instability
UTUC: Upper urinary tract urothelial carcinoma
